# Faking photon number on a transition-edge sensor

**DOI:** 10.1140/epjqt/s40507-022-00141-2

**Published:** 2022-09-05

**Authors:** Poompong Chaiwongkhot, Jiaqiang Zhong, Anqi Huang, Hao Qin, Sheng-cai Shi, Vadim Makarov

**Affiliations:** 1 Institute for Quantum Computing, University of Waterloo, Waterloo, ON N2L 3G1 Canada; 2 Department of Physics and Astronomy, University of Waterloo, Waterloo, ON N2L 3G1 Canada; 3 Department of Physics, Faculty of Science, Mahidol University, Bangkok, 10400 Thailand; 4 Quantum Technology Foundation (Thailand), Bangkok, 10110 Thailand; 5 Purple Mountain Observatory and Key Laboratory of Radio Astronomy, Chinese Academy of Sciences, 10 Yuanhua road, Nanjing, 210033 People’s Republic of China; 6 Institute for Quantum Information & State Key Laboratory of High Performance Computing, College of Computer Science and Technology, National University of Defense Technology, Changsha, 410073 People’s Republic of China; 7 Centre for Quantum Technologies, National University of Singapore, 3 Science Drive 2, Singapore, 117543 Singapore; 8 Russian Quantum Center, Skolkovo, Moscow, 121205 Russia; 9 Shanghai Branch, National Laboratory for Physical Sciences at Microscale and CAS Center for Excellence in Quantum Information, University of Science and Technology of China, Shanghai, 201315 People’s Republic of China; 10 NTI Center for Quantum Communications, National University of Science and Technology MISiS, Moscow, 119049 Russia

## Abstract

We study potential security vulnerabilities of a single-photon detector based on superconducting transition-edge sensor. In one experiment, we show that an adversary could fake a photon number result at a certain wavelength by sending a larger number of photons at a longer wavelength, which is an expected and known behaviour. In another experiment, we unexpectedly find that the detector can be blinded by bright continuous-wave light and then, a controlled response simulating single-photon detection can be produced by applying a bright light pulse. We model an intercept-and-resend attack on a quantum key distribution system that exploits the latter vulnerability and, under certain assumptions, able to steal the key.

## Introduction

Photon detectors are indispensable in quantum communication applications [1]. To ensure the reliability of detection results, it is important to characterize the detectors being used both within the intended working parameters and possible unintended conditions. This characterization could help in revealing possible flaws and imperfections. These flaws could lead to misguided detection results or, worse, exploitable vulnerabilities in the case of quantum cryptography applications. This characterization guides the work on improving the robustness of quantum systems. Over the years, many attacks have been reported on various types of photon detectors based on avalanche photodiodes [2–11] and superconducting nanowires [12–14]. This has led to the development of countermeasures [15, 16] and imperfection-insensitive protocols [17, 18].

Transition-edge sensor (TES) is a photon detector capable of providing full photon-number-resolving capability [19–21]. Optical TES arrays are under development and applied in few-photon color imaging [22–25]. A combination of TESes and lithium niobate waveguides makes available a variety of new quantum optics experiments [26]. The photon-number-resolving power of TES has been used for characterizing solid-state single-photon sources [27]. It has also achieved the highest detection efficiency among photon-number-resolving detectors up to 95% at $1550~\text{nm}$ [28–30]. This type of detector is used in various applications that require high detection probability, such as loophole-free Bell test [31]. Its photon number resolving capability could also be used to monitor against attacks on a quantum key distribution (QKD) system [32]. As one of the potential detectors in quantum communication where the reliability of detection result affects overall security, the TES photon detector should be investigated for its robustness and possible flaws. In this study, we experimentally demonstrate two potential vulnerabilities of TES, namely, a wavelength attack where the photon number result could be controlled by changing signal’s wavelength and a faked-state attack where the adversary increases the temperature of TES with an appropriate bright continuous-wave (CW) laser then forces an arbitrary photon number detection result using a bright pulsed laser.

## Experimental setup

**Figure 1:**
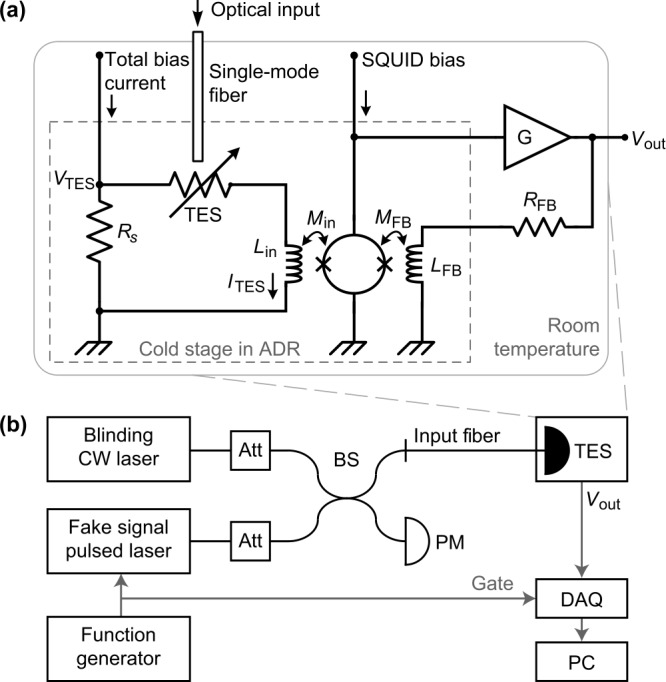
Experimental setup. (**a**) Internal circuit diagram of the TES system, consisting of the TES photon detector and its DC-SQUID readout. The TES photon detector is mounted on a 100-mK cold stage chilled by an adiabatic demagnetization refrigerator (ADR). The TES current $I_{\text{TES}}$ is readout by DC-SQUID electronics and transferred proportionally to a voltage output $V_{\text{out}}$. (**b**) Blinding and fake signal power is controlled by variable attenuators (Att), combined at a $50\!:\!50$ fiber-optic beam splitter (BS), measured by an optical power meter (PM), and applied to the TES system under test. Its output voltage $V_{\text{out}}$ is recorded and analyzed by a data acquisition module (DAQ) connected to a computer (PC)

A transition-edge sensor is a sensitive micro-calorimeter whose sensing element consists of an absorber and a superconductive thermometer with a positive temperature coefficient of resistance ($\mathrm{d}{R}/\mathrm{d}{T}>0$) [33]. During the operation, the sensing element’s temperature is kept near the transition temperature via voltage-biasing [34]. This voltage-biasing is provided by an external total bias current flowing through a shunt resistor $R_{s}$ connected in parallel with the TES [Fig. 1(a)]. In our setup $R_{s} = 16.1~\text{m}\Omega $, which is much smaller than the TES normal-conductivity resistance of $3~\Omega $.

The current passing through the TES $I_{\text{TES}}$ flows through an inductive coil $L_{\text{in}}$. The latter couples its magnetic flux via a mutual inductance ($M_{\text{in}}$) to a direct-current superconducting quantum interference device (DC-SQUID). The SQUID serves as a low-noise amplifier of $I_{\text{TES}}$. A feedback coil $L_{\text{FB}}$ inside the adiabatic demagnetization refrigerator (ADR), together with a room-temperature amplifier G and feedback resistor $R_{\text{FB}}$ are used to transform the signal from the TES into a measurable voltage $V_{\text{out}}$ [35]. $I_{\text{TES}}$ is obtained by dividing $V_{\text{out}}$ by the current-to-voltage gain of the DC-SQUID and amplifier G (0.375 V/μA in this experiment), while the voltage across TES $V_{\text{TES}}$ is calculated by multiplying $R_{s}$ by the current through it (total bias current with $I_{\text{TES}}$ subtracted).

When a photon from the input optical fiber hits the detector, the photon’s energy is absorbed, raising the TES’ temperature and resistance. This change of resistance reduces $I_{\text{TES}}$ and proportionally reduces $V_{\text{out}}$. From the relation of TES temperature and $I_{\text{TES}}$, it can be seen that the change of $V_{\text{out}}$ during the detection is proportional to the absorbed energy of the photon(s), enabling photon-number discrimination.

In our setup, the TES and SQUID are attached on a copper block attached in turn to the cold plate of the ADR. Under normal operating conditions, both the TES and SQUID are at $100~\text{mK}$ temperature. Their bias currents are provided by specialised electronic circuits (commercially available from Magnicon GmbH).

**Figure 2:**
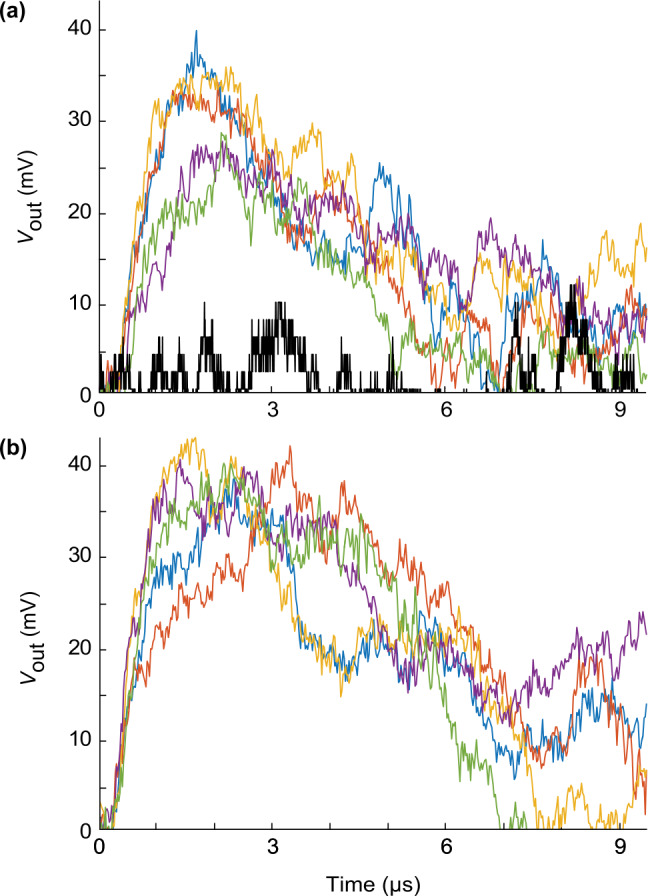
Oscillograms of $V_{\text{out}}$ without DC component. Each plot shows five typical oscillograms taken under identical conditions. (**a**) Single-photon responses (colored or gray traces). For comparison, a typical $V_{\text{out}}$ trace without an input pulse is plotted in black. (**b**) Fake “single-photon” responses under $1550~\text{nm}$ blinding attack with $2.4\times 10^{-18}~\text{J} $ pulse energy (i.e., about 19-photon weak coherent pulse)

To measure the response of the TES to various optical signals, we use a setup shown in Fig. 1(b). The TES is a fiber-coupled $10\times 10$ μm Ti device in a multilayer optical resonator designed to maximise coupling at $1550~\text{nm}$ wavelength and is similar to devices reported in [29, 36]. The photon coupling efficiency in our TES sample under test is ${\approx} 1\%$ owing to a misaligned fiber end to the TES effective area. However, this should not affect the results of our study in a qualitative way, because the misalignment merely introduces additional optical attenuation and can be compensated by applying a brighter test signal. Our light source consists of a CW blinding laser and a pulsed laser (with about $16~\text{ns} $ pulse width), combined on a fiber-optic beamsplitter (BS). The energy of laser pulse can be adjusted by the variable attenuator (OZ Optics DA-100). A power meter (PM) is used for monitoring the laser output power. A function generator produces trigger pulses to synchronize the laser source and signal recordings. The signal from the TES is digitized by a data acquisition module (DAQ) and analyzed on a computer (PC). The DAQ is a 16-bit, $125~\text{MHz} $ sampling rate analog-to-digital converter (AlazarTech ATS660) mounted on a peripheral component interconnect (PCI) bus of the PC. This DAQ allows measuring signals of millivolt level. Typical single-photon responses are shown in Fig. 2(a). These oscillograms show the signal without a constant bias (DC component), which is removed in post-processing. The peak voltage value during 5 μs following the application of the optical pulse is assumed to be the amplitude of the detector response $V_{\text{max}}$.

## Results

In this section, we investigate two potentially exploitable vulnerabilities of the TES detector.

### Wavelength-dependent response

TES output voltage amplitude $V_{\text{max}}$ is inherently proportional to the energy of photons absorbed, and sensitive to a wide range of wavelengths. In principle, *N* photons with a wavelength *Nλ* arriving simultaneously have the same combined energy E as one photon with the wavelength *λ*. This can be seen from the relation $E = N h c/\lambda $, where *h* is Planck’s constant and *c* is the speed of light in vacuum. Thus TES would produce the same output in these two cases [37–39]. This is a known property of the TES and a potential security vulnerability.

**Figure 3:**
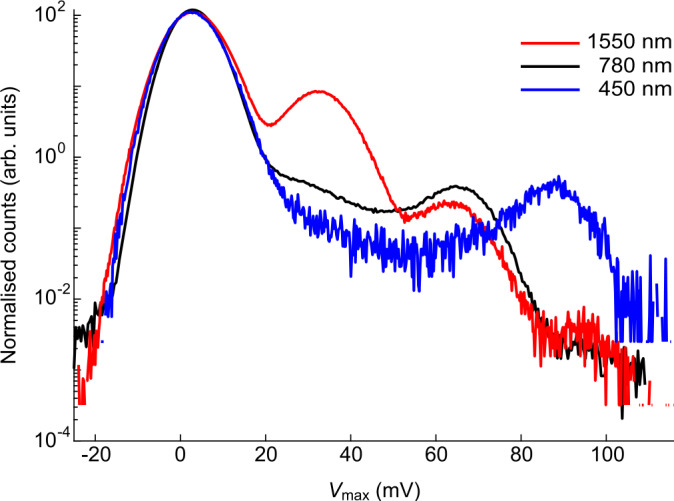
Histogram of TES output voltage under weak-coherent laser illumination at $1550~\text{nm}$ (red), $780~\text{nm}$ (black), and $450~\text{nm}$ (blue). The leftmost peak represents zero-photon detection. Subsequent peaks to the right represent higher photon number detections. These peaks appear at the voltage level proportional to the energy of the photons

We illustrate this fact with a simple experiment that shows how an attacker Eve could fake a single-photon detection result by sending multiple photons with proportionally lower photon energy. We send weak-coherent signals from several lasers of different wavelengths through the input fiber of the TES. We then record the voltage response’s amplitude $V_{\text{max}}$ from the TES. The histogram in Fig. 3 shows that the response signal of single-photon detection from a $450~\text{nm}$ photon is overlapped with two-photons detection from $780~\text{nm}$ and three-photons detection from $1550~\text{nm}$ photons. This shows that an expected photon number readout from the TES could be faked by multiple photons with a proportionally longer wavelength. It shows that the photon number measurement results from the TES alone cannot be used to characterize the photon number distribution of photon signal through an untrusted channel, such as the quantum channel, where the adversary could intercept and replace the signal with photons of arbitrary wavelength. Thus, any QKD scheme using photon number distribution from TES to monitor Eve’s activity in the quantum channel is vulnerable to this wavelength-dependent attack [32]. A narrow-band wavelength filter should prevent this attack. However, the characterization of the filter’s performance against exploitable wavelengths is needed.

### Blinding attack

In a blinding attack on QKD receiver, Eve renders the QKD detectors incapable of producing a typical single-photon detection result (blinded), but able to produce the expected detection output results when experiencing a bright-light pulse. This type of attack has been demonstrated in various single-photon detectors [4, 6–9, 12].

In the ideal condition, the TES operates at the transition edge between superconductivity and normal resistive state. In this region, a small change of energy such as single-photon absorption could induce a measurable change in the output voltage proportional to the energy absorbed. By setting a voltage threshold level for each input photon energy, one could discriminate the number of absorbed photons. From the known characteristic of TES [33] at a slightly higher temperature than the operational regime, it could produce the same voltage output level when absorbing much higher energy that can be delivered by a bright laser pulse. In this section, we experimentally demonstrate this behavior.

**Figure 4:**
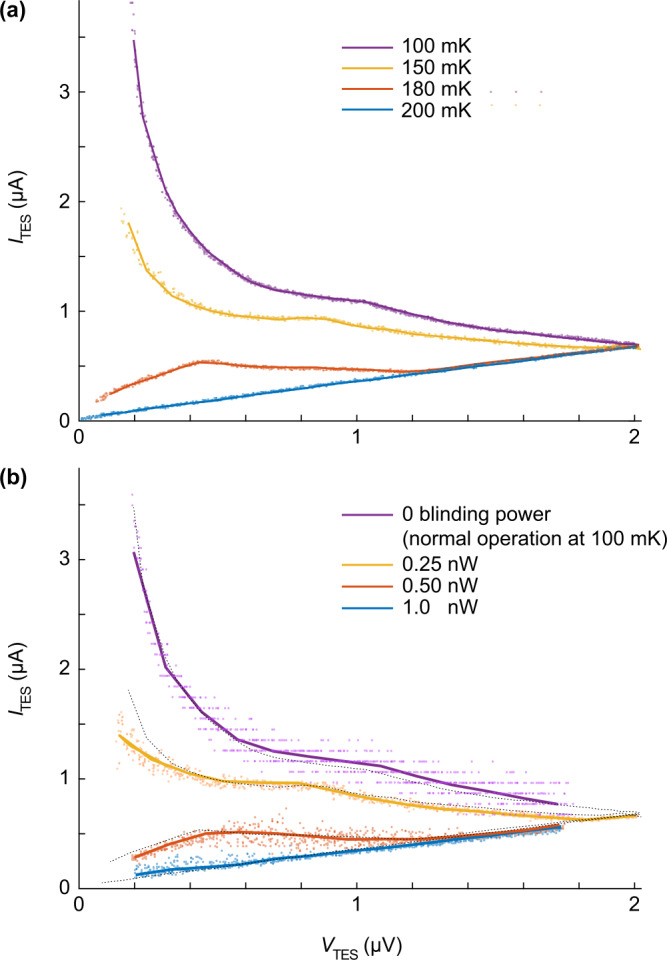
I–V curves of the TES (**a**) at different heat-bath temperatures and (**b**) at $100~\text{mK}$ temperature under bright laser illumination. Dots are measurement results while a solid line is their bin-averaging. For ease of comparison between the plots, the curves from (**a**) are reprinted as black dotted lines in (**b**); it can be seen that the plots closely resemble each other. This confirms Eve’s ability to control TES’s temperature using bright light through the input fiber

We first investigate the behavior of TES when its temperature is increased beyond the designed transition-edge region. We set the TES to the operating temperature of $100~\text{mK}$. We record the current-voltage (I–V) characteristic curves of the TES at different temperatures [29]. These characteristic curves, shown in Fig. 4(a), will be used as a reference for the following experiments. At low temperature ($100~\text{mK}$), $I_{\text{TES}}$ is roughly inverse proportional on $V_{\text{TES}}$. As the temperature increases, $I_{\text{TES}}$ becomes lower. Once the device reaches its critical temperature of $\approx 180~\text{mK}$, $I_{\text{TES}}$ becomes directly proportional on $V_{\text{TES}}$ as the TES becomes a normal resistor.

We now demonstrate the ability of Eve to control the temperature using bright light. A CW laser at $1550~\text{nm}$ is coupled through the input fiber of TES. Figure 4(b) shows that the I–V characteristics at different temperature of the device under test can be replicated. This shows that an adversary could arbitrarily control the temperature of TES using bright CW laser.

**Figure 5:**
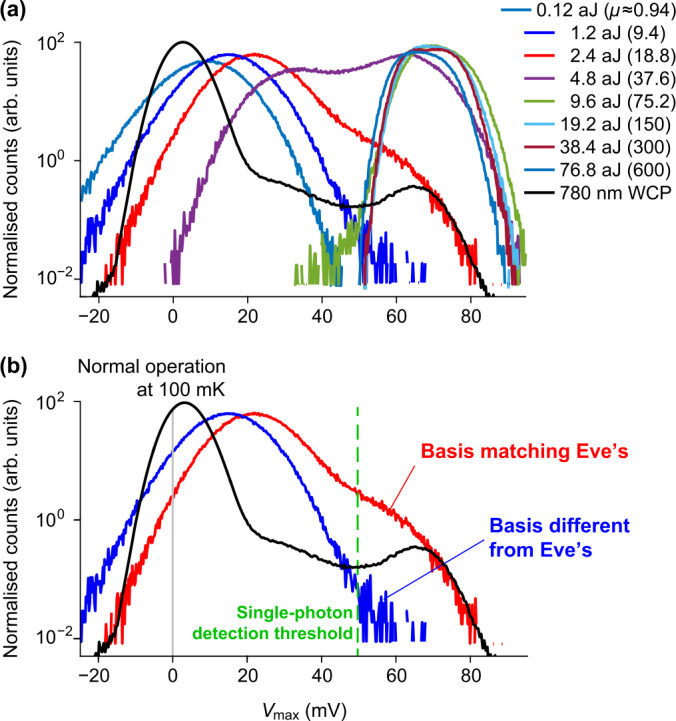
Detector response to the faked-state attack. For comparison, the black curve shows the normal response to a weak coherent pulse (WCP) attenuated to a single-photon level, containing the zero-photon response (left peak) and the one-photon response (right peak). (**a**) Fake histogram of output voltage at different faked-state pulse energies (their equivalent mean photon number *μ* is given in brackets). The detector is blinded with $0.25~\text{nW} $ CW light. (**b**) An attack model on a BB84 QKD system with TES as a detector. The threshold (green vertical dashed line) marks the minimum TES voltage output that the system in our model would register as a detection. The fake response is shown for two cases where Bob and Eve pick the same (red) and different (blue) measurement bases under fake pulsed signal of $2.4\times 10^{-18}~\text{J} $ pulse energy

For the faked-state attack, the appropriate blinding laser power is one that puts the response at the threshold between the transition-edge regime and the normal resistor regime. In this region, the TES is ‘blinded’ from single-photon input as the change of voltage produced by an additional absorption is minimal. At the same time, the system in this condition could produce the same voltage level as the system at normal operating temperature when absorbing a bright laser pulse. The histogram of faked-state results with different peak power is shown in Fig. 5(a) and typical oscillograms in Fig. 2(b). Here, the fake signals are laser pulses with $16~\text{ns} $ width and $100~\text{kHz} $ repetition rate (i.e., 10 μs interval). The histograms correspond to the detection within the first 5 μs window that is equivalent to half the interval between the consecutive pulses. We did this to reduce background counts. We record ≈3300 samples for each histogram. The detector response exhibits a strong superlinearity [40] between Eve’s pulse energies of 1.2 to $9.6 \times 10^{-18}~\text{J} $ (mean photon number $\mu \approx 9$ to 75). This is a potential security loophole, i.e., the voltage response of TES can be controlled by Eve who has access to the input channel. She can choose a bright laser power such that the voltage output represents a ‘photon number’ of her choice.

The physics of the detector in this regime is not clear to us and needs to be investigated further. Multiplying the mean photon number by the assumed coupling efficiency (1%) yields much less than one photon per pulse in the region of strong superlinearity, which cannot explain the observed drastic change in the distributions in Fig. 5(a). A follow-up experiment with another TES sample may be needed.

### Attack model

To emphasize the threat of vulnerability found in the previous section, we pick a well-known attack on a well-known QKD protocol. Although the TES has the photon number resolving capability and may be used in future implementations of more advanced protocols, we assume for simplicity that it is used as a threshold detector in the standard Bennett-Brassard 1984 (BB84) [41] QKD system. Here we model a faked-state attack on this system [4]. We assume here that the wavelength of the signal used by Alice and Bob is $780~\text{nm}$. In this attack model, the adversary Eve intercepts each signal from Alice and measures it in a random basis. She then reproduces a bright fake signal identical to her detection result and sends it to Bob. Here, she also sends a CW blinding laser power set to $0.25~\text{nW} $ and sets her fake pulsed signal at $2.4\times 10^{-18}~\text{J} $ pulse energy, both at $1550~\text{nm}$. In case of Bob’s measurement basis choice being different from that of Eve, the power of the fake signal would be split equally between Bob’s detectors (we assumed here Bob’s basis choice modulator is wavelength-independent). As shown in Fig. 5(b), most of the response signal from TES would fall below the single-photon detection threshold, thus remain unregistered. However, if their basis choices matched, sometimes the signal will be registered. This attack condition causes extra detection loss in Bob. Eve could hide this loss from Alice and Bob if the original quantum channel loss between Alice and Bob is lower than the detection loss induced by Eve’s attack. When the basis of measurement between Eve and Bob are different, half of the registered detection events would cause an error in the key. This can be seen in the portion of the blue histogram to the right of the single-photon threshold (green line) in Fig. 5(b). With this estimated detection probability and error rate, the quantum bit error rate of the attack could be calculated. Our calculation shows that this attack on a QKD system with the TES under test and the specific parameters assumed above would induce 7.4% quantum bit error rate (QBER). This QBER is lower than the 11% abort threshold of the BB84 protocol [42], thus the security of the key could be compromised.

This shows a possible vulnerability of a QKD system with TES as a single-photon threshold detector. This attack is applicable to other QKD protocols that use threshold detectors, such as coherent-one-way (COW) protocol [7]. For future QKD schemes that use the TES as a photon number resolving detector, an attack with multiple detection thresholds will need to be constructed.

## Conclusion

We have experimentally demonstrated two possible security vulnerabilities of TES as a photon detector. In this study, we have illustrated the ability of Eve to fake photon-number results in TES using different wavelengths. We have also shown that the characteristics of TES could be altered by a bright CW laser, and photon-number detection results could be faked using laser pulses with appropriate peak power. Using this result, we model an attack on a BB84-QKD system with TES as a detector and show that Eve could perform the intercept-and-resend attack while inducing as low as 7.4% error rate, under certain specific assumptions. Since the TES under test has a misalignment of its input coupling, which limits its detection efficiency, we speculate that an attack on a higher-efficiency TES with better energy resolution might yield a better result for Eve. Understanding a physical model of the TES under attack can be a topic of a future study. Countermeasures to such attacks, such as adding a pulse integrator to detect the changes in the output voltage’s pulse shape, will need to be considered in the future when TESes begin getting employed in secure quantum communication schemes. However, the effect on performance and possible loopholes of each countermeasure will need a further investigation.

## Data Availability

Raw experimental data and calculations can be obtained from the corresponding author upon a reasonable request.

## Abbreviations

TES: transition-edge sensor
QKD: quantum key distribution
CW: continuous-wave
DC-SQUID: direct-current superconducting quantum interference device
ADR: adiabatic demagnetization refrigerator
DAQ: data acquisition unit
QBER: quantum-bit error rate
BB84: Bennett-Brassard 1984 (QKD protocol)
COW: coherent-one-way (QKD protocol)
